# Climate shaped the worldwide distribution of human mitochondrial DNA sequence variation

**DOI:** 10.1098/rspb.2009.0752

**Published:** 2009-07-08

**Authors:** François Balloux, Lori-Jayne Lawson Handley, Thibaut Jombart, Hua Liu, Andrea Manica

**Affiliations:** 1 Department of Infectious Disease Epidemiology, Imperial College Faculty of Medicine, MRC Centre for Outbreak Analysis and Modelling, St Mary's Campus, Norfolk Place, London W2 1PG, UK; 2 Department of Biological Sciences, University of Hull, Cottingham Road, Hull HU6 7RX, UK; 3 Department of Genetics; 4 Department of Zoology, University of Cambridge, Downing Street, Cambridge CB2 3EJ, UK

**Keywords:** mtDNA, selection, climate, temperature, human evolution, single nucleotide polymorphisms

## Abstract

There is an ongoing discussion in the literature on whether human mitochondrial DNA (mtDNA) evolves neutrally. There have been previous claims for natural selection on human mtDNA based on an excess of non-synonymous mutations and higher evolutionary persistence of specific mitochondrial mutations in Arctic populations. However, these findings were not supported by the reanalysis of larger datasets. Using a geographical framework, we perform the first direct test of the relative extent to which climate and past demography have shaped the current spatial distribution of mtDNA sequences worldwide. We show that populations living in colder environments have lower mitochondrial diversity and that the genetic differentiation between pairs of populations correlates with difference in temperature. These associations were unique to mtDNA; we could not find a similar pattern in any other genetic marker. We were able to identify two correlated non-synonymous point mutations in the *ND3* and *ATP6* genes characterized by a clear association with temperature, which appear to be plausible targets of natural selection producing the association with climate. The same mutations have been previously shown to be associated with variation in mitochondrial pH and calcium dynamics. Our results indicate that natural selection mediated by climate has contributed to shape the current distribution of mtDNA sequences in humans.

## 1. Introduction

Mitochondrial DNA (mtDNA) remains by far the most widely used genetic marker in studies of human populations. One assumption behind inferences on past human demographic history is the selective neutrality of the genetic markers employed. There have been claims for natural selection affecting mtDNA, with temperature being highlighted as a possible selective force in a variety of taxa (Ballard & Whitlock 2004) including humans (Torroni *et al*. 2001; Mishmar *et al*. 2003; Ruiz-Pesini *et al*. 2004). However, this has been rejected by several studies, which concluded that human mtDNA sequence variation has not been significantly shaped by climate (Elson *et al*. 2004; Kivisild *et al*. 2006; Amo & Brand 2007; Ingman & Gyllensten 2007; Sun *et al*. 2007). The tests so far have mainly relied on ratios of synonymous and non-synonymous mutations (*d*N/*d*S ratios) and to a lesser extent on the evolutionary persistence of mutations in the mitochondrial phylogenetic tree. Interestingly, it has since been shown that *d*N/*d*S ratios are largely inadequate when testing for natural selection within populations (Kryazhimskiy & Plotkin 2008). Here we take a radically different approach by directly modelling the distribution of worldwide mitochondrial sequence diversity with geography and climatic variables.

The most likely origin of anatomically modern humans lies in sub-Saharan Africa, where the most ancient remains (dated to approximately 200 000 years) have been found (McDougall *et al*. 2005). It is generally accepted that the human population started expanding its range 50 000–70 000 years ago and then colonized the entire globe with little or no interbreeding with previously established archaic human species (Stringer & Andrews 1988; Macaulay *et al*. 2005; Liu *et al*. 2006; Fagundes *et al*. 2007; Hellenthal *et al*. 2008; Deshpande *et al*. 2009). A signature of this expansion can be seen in the smooth clinal geographical distribution of autosomal polymorphisms (Handley *et al*. 2007). Genetic differentiation between populations increases essentially linearly with geographical distance along landmasses (Relethford 2004; Manica *et al*. 2005; Ramachandran *et al*. 2005; Romero *et al*. 2008) and geographical distance from sub-Saharan Africa is an excellent predictor of the genetic diversity of individual populations throughout the world (Prugnolle *et al*. 2005*a*). We can capitalize on these exceptionally strong correlations between genetics and geography by using physical distance as a proxy for past demography (Prugnolle *et al*. 2005*b*). This in turn allows for formal statistical testing of the extent to which demography and climate have shaped the current distribution of mitochondrial sequence diversity.

## 2. Material and methods

### (a) Data

**Figure 1.**
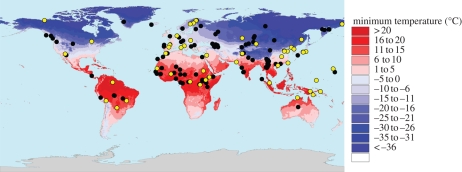
Map of locations of populations from which HVS-I sequences (black) and complete mtDNA genomes (yellow) were obtained. The background colour gradient (blue to red) represents the minimum temperature.

We downloaded over 5000 hyper-variable segment I (HVS-I) sequences from the HVRBase++ online resource (Kohl *et al*. 2006). Only non-admixed native populations were considered and we enforced a minimal sample size of 10. This led to the inclusion of 109 populations providing a good worldwide cover (figure 1; electronic supplementary material, table S1). When more than 100 HVS-I sequences were available for a sample, we randomly selected 100 sequences. The sample size per population ranged between 10 and 100 individuals, with a mean and median number of 47 and 42 per population, respectively. All HVS-I sequences were aligned with ClustalX v. 2.0.9 and double-checked visually. The alignment covered the entire HVS-I region (positions 16 024–16 365 relative to the Cambridge Reference Sequence, CRS). The poly-C tract located between positions 16 184 and 16 193 proved difficult to align unambiguously and had not been successfully amplified in a substantial fraction of available sequences. Thus, this section was removed from all analyses.

Full genome sequences were downloaded from the Human Mitochondrial Genome Database (mtDB; Ingman & Gyllensten 2006) and from GenBank (figure 1; electronic supplementary material, table S2). The HVS-I and HVS-II sequences were excised. The selection criteria were identical to those for the HVS-I dataset but, owing to the lower availability of complete genomes, we enforced a lower minimal sample size of five individuals per populations. In addition, we did not include any individual we knew to have been sequenced based on prior knowledge of its mitochondrial haplotype. We were able to retrieve 628 sequences comprising 51 populations (median sample size = 8).

All autosomal (783), X- and Y-chromosome microsatellites (36 and 6, respectively) were downloaded from the HGDP-CEPH human genome diversity panel database v. 2.0 (http://www.cephb.fr/en/hgdp/). The genetic diversity for single nucleotide polymorphisms (SNPs) treated as phased haplotypes were obtained from table S1 in the electronic supplementary material in Li *et al*. (2008). The analyses were performed on the H971 subset of the HGDP-CEPH panel (Rosenberg 2006), which excludes duplicates, misassigned samples and suspected first-degree relative pairs. To ensure consistency between the various genetic markers typed on the HGDP-CEPH, we removed the two small heterogeneous Southern Bantu populations. All the information on the HGDP-CEPH panel populations is summarized in table S3 of the electronic supplementary material.

### (b) Geographical analyses

We assigned geographical coordinates to every population based on the information in the original articles. As very few authors provide sample coordinates, we had to rely on the names of the localities given or in some cases on the maps provided in the papers. Minimum temperature was obtained from WORLDCLIM (Hijmans *et al*. 2005), as sets of global climatic GIS layers with a 30 arc-seconds resolution. Geographical distances were computed as shortest distances along landmasses, avoiding mountain regions with average altitude over 2000 m. These were obtained within a spherical referential using graph theory (Manica *et al*. 2005; Prugnolle *et al*. 2005*a*). The hypothetical origin for anatomically modern humans was chosen as −12° latitude and 25° longitude, as this set of coordinates has been previously shown to represent the most likely location based on genetic and morphological data (Manica *et al*. 2007).

### (c) Genetic diversities

All genetic diversities and genetic distances for mtDNA used in the paper were computed as average pairwise difference in Arlequin v. 3.1 (Excoffier *et al*. 2005) assuming a Tamura and Nei 93 model of sequence evolution (Tamura & Nei 1993). The probabilities of transitions and transversions were estimated from the data using Arlequin (Excoffier *et al*. 2005). The gamma shape parameter *α* was set to 1.12 for the whole genomes as estimated using Modeltest 3.6 (Posada & Crandall 1998) and to 0.40 for the HVS-I sequences following previous recommendation (Excoffier & Yang 1999). Summary statistics for the autosomal and X- and Y-chromosome polymorphism were obtained with Fstat v. 2.9.3 (Goudet 1995).

To test the effect of past demography (distance from sub-Saharan Africa) and climate (minimum temperature), we built a linear model with within-population genetic diversity as the response variable, and with distance and temperature as predictors. The previous analysis assumes independence between populations and may thus overestimate degrees of freedom. Thus, we replicated all analyses based on genetic diversity with Mantel and partial Mantel tests. The different matrices were computed as pairwise difference in genetic diversity, absolute difference in minimum temperature and absolute difference in the geographical distance from the hypothetical sub-Saharan origin. The results from the Mantel tests were qualitatively similar to the ones obtained with the linear models and are reported in table S4 in the electronic supplementary material.

We also tested whether there may be an effect of sample size. There was no significant correlation between the number of individuals typed per population and genetic diversity for HVS I sequences (*R*^2^ = 0.006, *F*_1,107_ = 1.688, *p* = 0.197) or complete mitochondrial genomes (*R*^2^ < 0.001, *F*_1,49_ = 0.819, *F*_1,107_ = 1.692, *p* = 0.370). To test whether the association between mtDNA and climate might have arisen by chance, we compared the proportion of variance in mtDNA diversity explained by climate to the same measure estimate for each of the 783 autosomal STRs individually. All pairwise correlations were computed with Mantel tests and partial Mantel tests.

### (d) Candidate single nucleotide polymorphisms

For the analysis of candidate targets for natural selection, we recovered all polymorphisms with a minor allele frequency of 10 per cent or higher and computed their frequency in all 51 populations for which we had complete genome information. The mutations comprised 32 point mutations and two InDels. We used the MITOMAP database (Ruiz-Pesini *et al*. 2007) to identify synonymous and non-synonymous SNPs. Geographical distance from a hypothetical source in an isolation-by-distance model cannot be used as a predictor for the frequency of single alleles. In order to disentangle the effect of climate and past demography, we relied on a partial Mantel test to first quantify the association between differences in frequency of a given SNP and geographical distance between two populations, and then test for an effect of temperature difference above and beyond isolation by distance. Euclidean distances in SNP frequencies were used in the matrix of pairwise genetic distances. Once we had recovered the two SNPs, which showed an association with climate after isolation by distance had been accounted for, we modelled their association with climate. This was done by building a generalized linear model of the frequency of the given SNPs within each population as predicted by minimum temperature, with a binomial error structure and a logit link to account for the fact that each individual can only either have or not have the focal allele. We considered the proportion of explained deviance as it is logically analogous to the proportion of variance (*R*^2^) adopted for other models.

A phylogenetic tree of the complete mitochondrial genomes was obtained by neighbour joining of a matrix of pairwise genetic distances between DNA sequences (Tamura & Nei 1993). One hundred bootstraps were performed based on resampling nucleotides. The ancestral or derived state was mapped on the phylogeny for the SNPs correlated to minimum temperature using the APE package (Paradis *et al*. 2004).

## 3. Results and discussion

**Figure 2.**
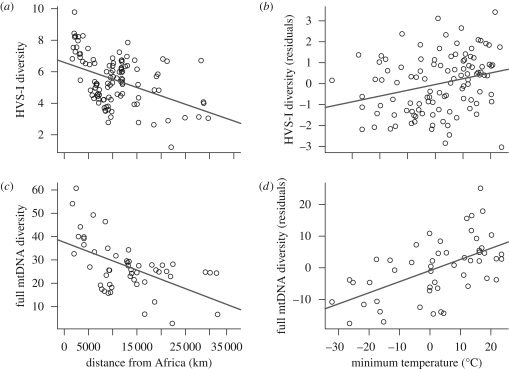
Relationship between mtDNA within-population genetic diversity, distance from sub-Saharan Africa and minimum temperature: (*a*) genetic diversity for mitochondrial HVS-I sequences against distance from Africa (km); (*b*) genetic diversity for mitochondrial HVS-I sequences against minimum temperature, after correcting for distance from Africa; (*c*) genetic diversity for complete mitochondrial genome sequences against distance from Africa (km); and (*d*) genetic diversity for complete mitochondrial genome sequences against minimum temperature, after correcting for distance from Africa.

Distance from Africa along landmasses explains 18.3 per cent of the variance in within-population genetic diversity for the first hypervariable segment of mtDNA (HVS-I) from 109 populations distributed worldwide (*F*_1,107_ = 25.2, *p* < 0.001; figure 2*a*). This figure is low compared with the 84.9 per cent reported for the 54 HGDP-CEPH populations typed at 783 microsatellites (Handley *et al*. 2007). It is also lower than the amount of phenotypic variation based on craniometric traits explained by distance from Africa (von Cramon-Taubadel & Lycett 2008; Betti *et al*. 2009). HVS-I genetic diversity is also significantly associated with minimum temperature, which explains 7.8 per cent of variance (*F*_1,107_ = 10.2, *p* = 0.002). Since sub-Saharan Africa comprises some of the warmest areas on earth, it might be expected that minimum temperature and distance from Africa are related. In fact, distance from Africa and minimum temperature for the sampled populations are not correlated (*R*^2^ = 0.010, *F*_1,107_ = 1.3, *p* = 0.254). Even when we accounted for the effect of distance from Africa, the relationship between HVS-I diversity and minimum temperature remained significant (*R*^2^ = 0.055, *F*_1,106_ = 8.7; *p* = 0.004, figure 2*b*). Thus, both distance from Africa and low temperatures decrease within-population mtDNA diversity at HVS-I.

Does climate also influence population differentiation as measured with HVS-I sequences? To test for this, we produced matrices for between-population pairwise genetic distances, geographical distances along landmasses and difference in minimum temperature. When we ran separate Mantel tests, we obtained correlations of *r*_M_ = 0.47 (*p* < 0.001; electronic supplementary material, fig. S1*a*) between genetic and physical distance, and *r*_M_ = 0.22 (*p* < 0.001; electronic supplementary material, fig. S1*b*) between genetics and temperature. Testing for an effect of temperature after accounting for geographical distance with a partial Mantel confirms the correlation with temperature (*r*_M_ = 0.20; *p* < 0.001). While the correlation with climate is moderate, this effect is expected to colour analyses of population differentiation at large geographical scales.

To confirm that these patterns were not specific to the HVS-I sequences, we repeated the analyses above on a smaller dataset of complete mitochondrial sequences from 51 populations with HVS-I and HVS-II regions removed (figure 1; electronic supplementary material, table S2). We recovered even more striking results: the decrease in genetic diversity with distance from Africa is slightly higher (*R*^2^ = 0.293, *F*_1,49_ = 20.45, *p* < 0.001; figure 2*c*), and climate explains a far higher amount of variance (*R*^2^ = 0.210, *F*_1,49_ = 14.3, *p* < 0.001; after correcting for distance from Africa: *R*^2^ = 0.215; *F*_1,48_ = 21.8, *p* < 0.001; figure 2*d*). We also confirm that genetic differentiation between populations correlates with geographical distances (*r*_M_ = 0.25, *p* = 0.011; electronic supplementary material, fig. S1*c*) and differences in minimum temperature (*r*_M_ = 0.17, *p* = 0.020; electronic supplementary material, fig. S1*d*; partial Mantel after correcting for climate: *r*_M_ = 0.16, *p* = 0.026).

**Figure 3.**
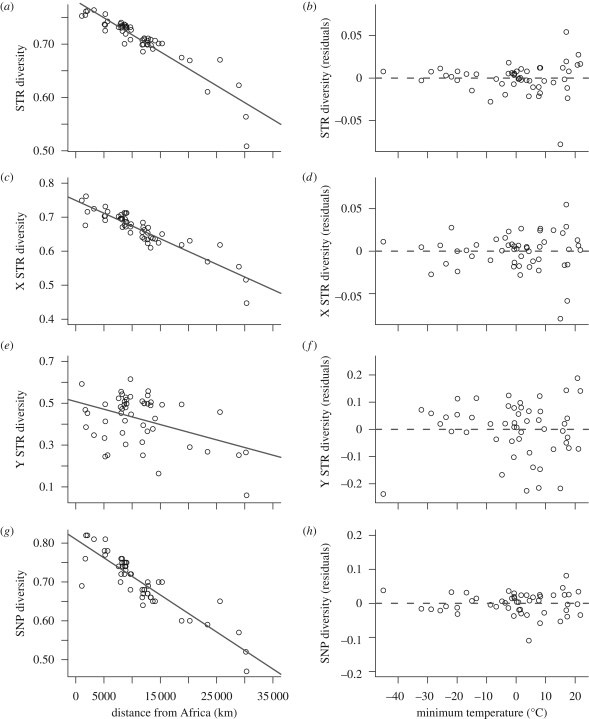
Relationship between within-population genetic diversity, distance from sub-Saharan Africa and minimum temperature: genetic diversity against distance from Africa (km) at (*a*) autosomal microsatellites, (*c*) autosomal SNPs, (*e*) X-chromosome microsatellites and (*g*) Y-chromosome microsatellites; genetic diversity against minimum temperature, after correcting for distance from Africa, at (*b*) autosomal microsatellites, (*d*) autosomal SNPs, (*f*) X-chromosome microsatellites and (*h*) Y-chromosome microsatellites.

A potential explanation for the association between climate and the distribution of mitochondrial sequence variation could stem from populations in colder environments being smaller and more isolated. If this is the case, a similar demographic effect should be picked up by other genetic markers. To tackle this issue, we first tested whether minimum temperature was also a predictor for the mean genetic diversity of autosomal microsatellites in the HGDP-CEPH panel. While microsatellite diversity was strongly influenced by distance from Africa (*R*^2^ = 0.849, *F*_1,49_ = 238.1, *p* < 0.001; figure 3*a*), we detected no effect of climate (*R*^2^ < 0.001, *F*_1,48_ = 1.0, *p* = 0.329; figure 3*b*). mtDNA is haploid and inherited through the female line only without any genetic recombination. As such, it can be more sensitive to population bottlenecks (Fay & Wu 1999) and subject to high stochasticity in allele frequencies, which is averaged out on the autosomal datasets owing to the large number of markers deployed. We thus tested whether such an association with climate could easily be obtained for individual microsatellites, which are believed to be neutral. The strength of the association between climate and mtDNA within-population diversity was significantly higher than for individual microsatellites (*p* = 0.010; electronic supplementary material, fig. S2), giving further support to a direct link between climate and within-population mitochondrial diversity.

As autosomal microsatellites are characterized by a far higher mutation rate than mtDNA sequences, the signature of stronger bottlenecks in colder regions may have been partly erased by subsequent mutations replenishing the genetic diversity of populations from cold areas. We thus replicated the analysis on the mean heterozygosity of haplotypes constructed from phased autosomal SNPs (Li *et al*. 2008) characterized by an even lower mutation rate than mtDNA sequences. Again, we found a strong effect of distance from Africa (*R*^2^ = 0.802, *F*_1,49_ = 204.1, *p* < 0.001; figure 3*c*), but no signal for climate (*R*^2^ < 0.001, *F*_1,48_ = 0.5, *p* = 0.477; figure 3*d*). The absence of any association with temperature for both microsatellites and SNPs argues against a spurious effect mediated by differences in mutation rates between classes of genetic markers.

Owing to its maternal inheritance, mtDNA is only affected by the past demography of the female line. For example, an association between climate and mitochondrial genetic diversity could arise if the ratio of male over female immigration was correlated with temperature. The same pattern could be generated if sex-specific variances in reproductive success covaried with climate. To test for such an effect, we repeated our analysis on the genetic diversity of 36 X-chromosome microsatellites typed in the HGDP-CEPH panel. Females transmit an X chromosome to both their daughters and sons, whereas males pass on their single copy to their daughters only. Thus, X-chromosome markers are more strongly affected by the past demography of the female line. Apart from this difference, autosomal and X-chromosome microsatellites should be largely comparable as both undergo recombination and do not seem to be characterized by significantly different mutation patterns in humans (Xu *et al*. 2005). Thus, looking at X-chromosome markers provides a way to detect any association between climate and mating system or sex-biased dispersal. Genetic diversity at the X-chromosome microsatellites decreases with increasing distance from Africa (*R*^2^ = 0.827, *F*_1,49_ = 240.0, *p* < 0.001; figure 3*e*), but, importantly, there was no trace of a signal from climate (*R*^2^ = 0.004, *F*_1,48_ = 2.15, *p* = 0.149; figure 3*f*).

The Y chromosome is only inherited down the paternal line, and thus constitutes a male analogue of mtDNA. If the effect we find on mtDNA is a feature of its lack of recombination, uniparental inheritance or small effective population size (relative to the autosomes and X chromosome), we would therefore expect to find a similar pattern on the Y chromosome. To investigate this, we tested whether diversity in microsatellites on the Y chromosome showed similar patterns to the rest of the genome. We found a significant decline in diversity with increasing distance from Africa (*R*^2^ = 0.164, *F*_1,49_ = 10.8, *p* = 0.002; figure 3*g*) and no relationship with minimum temperature (*R*^2^ = 0.013, *F*_1,48_ = 1.7, *p* = 0.193; figure 3*h*), mirroring the results for autosomal and X-chromosome diversity. Having markers that are either passed down the male line or predominantly the female line provides us with a powerful test for an association between climate and mating system or sex-biased dispersal, which would be highlighted by a link between climate and the ratio of diversities at the two uniparental markers. When we modelled the distribution of ratios of diversity at the X and Y chromosomes, we found no effect of minimum temperature (*R*^2^ < 0.001, *F*_1,49_ = 0.9, *p* = 0.351). This result indicates that minimum temperature does not seem to induce any systematic variation in mating system or sex-specific migration that could have created a spurious association between climate and mtDNA sequence distribution.

We next asked whether the relationship between mtDNA and climate may be driven by greater representation of Arctic populations in the mtDNA datasets, relative to the HGDP-CEPH panel. To test for this possibility, we removed populations one by one from the larger HVS-I dataset, starting with the population from the coldest location and moving to progressively warmer climates, until we had removed all populations with a minimum temperature below 15°C. The correlation between mtDNA diversity and distance from Africa increased steadily from 18.3 per cent to about 70 per cent as populations were removed (electronic supplementary material, fig. S3). This smooth increase illustrates that the correlation with climate is not driven by a few populations from extreme environments but acts over a broad range of temperatures including the entire temperate zone. Indeed, the increase in the correlation with geography even extends to the 10–15°C minimum temperature bracket, which comprises areas such as parts of East Africa or large swathes of Southeast Asia, which are all regions that are believed to have been inhabited for a long time and have maintained sizeable human populations.

**Figure 4.**
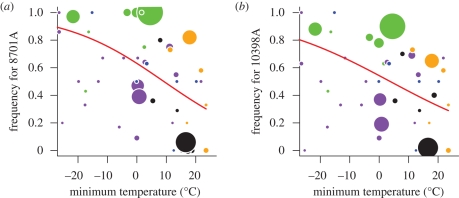
Association between minimum temperature and allele frequency at two non-synonymous mitochondrial SNPs: (*a*) 8701 and (*b*) 10398. The frequency of the derived A nucleotide was plotted in both panels. The size of the points is proportional to the sample size of the various populations. African populations are represented in black, European in green, Asian in purple, American in blue and Oceanian in orange.

In the next step, we searched for specific nucleotides in our whole genome mtDNA dataset that may be the direct targets of selection. We retrieved all polymorphisms with a minor allele frequency of 0.1 or higher. We detected 34 such sites, including 32 SNPs and two insertion/deletion variants. Five of the SNPs represented non-synonymous changes (Ruiz-Pesini *et al*. 2007). To test for any effect of selection by temperature on individual SNPs, we first needed to correct for isolation by distance. Neighbouring populations are exposed to similar climates but will also be characterized by similar allele frequencies due to recent common ancestry and gene flow. To disentangle demography and climate, we used a partial Mantel test that first quantifies the link between differences in frequency of a given SNP and geographical distance between two populations, and then tests for an effect of temperature difference above and beyond isolation by distance. When we ran partial Mantel tests on the five SNPs, only two showed a significant link with minimum temperature: 8701G/A (relative to the CRS), which lies in the *ATP6* gene (*r*_M_ = 0.11, *p* = 0.011), and 10398G/A in the *ND3* gene (*r*_M_ = 0.14, *p* = 0.005). The allele frequencies at these two SNPs are tightly linked (*R*^2^ = 0.862; average of the other pairwise comparisons 
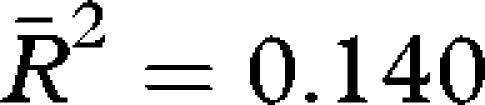
). When we plotted the allele frequency in individual populations against minimum temperature, there was a significant relationship for both SNPs, with temperature explaining 16.8 per cent (*χ*^2^_1_ = 74.7, *p* < 0.001; figure 4*a*) of the variance for 8701G/A and 15.3 per cent (*χ*^2^_1_ = 51.2, *p* < 0.001; figure 4*b*) for 10398G/A. For both polymorphisms, the G nucleotide is fixed or nearly fixed in sub-Saharan populations. The Americas show a similar gradient to the one found in Eurasia: the A nucleotide is predominant in Arctic populations close to the Bering Strait (the entry point to the Americas) and becomes less frequent further south. This leads to a strong correlation between minimum temperature and the two SNPs even when Beringian and American populations are considered in isolation (8701G/A: *R*^2^ = 33.9, *χ*^2^_1_ = 8.4, *p* = 0.004; 10398G/A: *R*^2^ = 39.5, *χ*^2^_1_ = 9.5, *p* = 0.002).

Because the G nucleotides at both SNPs are nearly fixed in Africa and largely absent from Europe, one may argue that the effect is solely driven by these two continents. However, even after removing all African populations, we still find a significant link with climate for both SNPs (8701G/A: *R*^2^ = 0.035, *χ*^2^_1_ = 8.4, *p* = 0.004; 10398G/A: *R*^2^ = 0.038, *χ*^2^_1_ = 6.7, *p* = 0.009), confirming that our results are not simply due to the loss of some haplotypes during an out-of-Africa bottleneck. Europe has been argued to have been recolonized only relatively recently, following the last glacial maximum. Thus, we also removed all European populations to test whether very recent bottlenecks during this period might be responsible for the association with climate. Again, the link with climate remained significant after all European populations were excluded (8701G/A: *R*^2^ = 0.112, *χ*^2^_1_ = 74.7, *p* < 0.001; 10398G/A: *R*^2^ = 0.084, *χ*^2^_1_ = 16.3, *p* < 0.001). As the association between the two SNPs and climate is robust to the elimination of all African or European populations, we can conclude that it is a global pattern.

**Figure 5.**
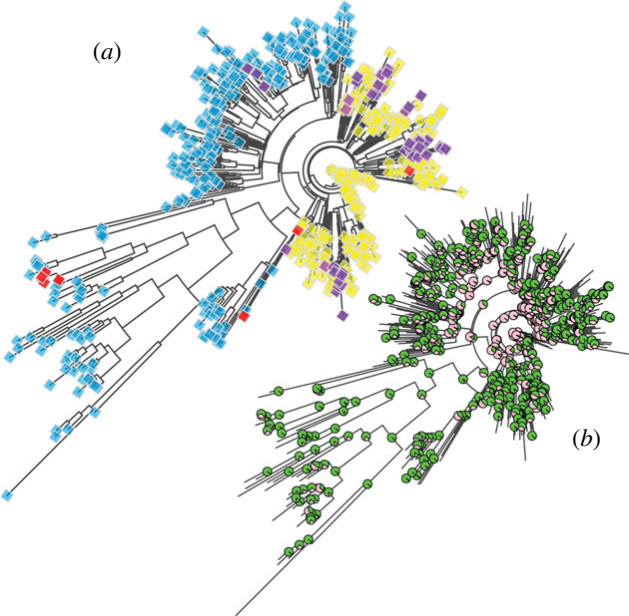
Phylogenetic tree for the complete mitochondrial genome dataset. (*a*) Ancestral and derived states for the 8701G/A and 10398G/A SNPs. Blue, SNP 8701 = ‘G’, SNP 10398 = ‘G’; yellow, SNP 8701 = ‘A’, SNP 10398 = ‘A’; violet, SNP 8701 = ‘A’, SNP 10398 = ‘G’; red, SNP 8701 = ‘G’, SNP 10398 = ‘A’. (*b*) Bootstrap support values are indicated by pie charts with supported partitions displayed in green and unsupported ones in pink.

While we document strong geographical patterns in the distribution of mtDNA suggestive of natural selection driven by climate, we have not addressed the underlying selective dynamics. The two possible extreme scenarios are purifying selection in cold geographical regions on the standing mitochondrial variation, or, alternatively, selective sweeps on new mutations in harsher climate. To test for this, we built a phylogenetic tree on the complete mitochondrial dataset and mapped the allelic state for the two candidate SNPs on all terminal branches (figure 5). The distribution of the two SNPs in the phylogeny suggests a scenario where an important selective sweep generated a large clade where haplotypes with the derived alleles predominate. However, we also observe multiple secondary reversals to the ancestral state, in particular for 10398G/A (figure 5). Interestingly, the 10398 site has previously been identified by Kivisild *et al*. (2006) as a likely target of natural selection and they inferred seven substitutions at this site within their 277 genomic sequences.

The 10398G/A polymorphism has been linked to longevity (Niemi *et al*. 2005) and a variety of neurological disorders, such as Parkinson's disease, Alzheimer's disease or bipolar disorder (Kato *et al*. 2001; van der Walt *et al*. 2003, 2004). The same polymorphism has been reported to be involved in invasive breast cancer and prostate cancer in African-Americans (Canter *et al*. 2005; Mims *et al*. 2006) and type 2 diabetes in North Indians (Rai *et al*. 2007). The two SNPs we found to be associated with temperature were also identified as affecting mitochondrial matrix pH and mitochondrial calcium dynamics (Kazuno *et al*. 2006). The 8701G/10398G haplotypes dominant in populations from warm environments were shown to have a higher mitochondrial matrix pH and lower intra-mitochondrial calcium levels (Kazuno *et al*. 2006), which may reduce the electrochemical gradient that drives ATP synthesis. 10398G/A corresponds to an alanine amino acid residue being substituted by a threonine at the C terminus of *ND3*, a subunit of NADH-ubiquinone oxidoreductase (complex I). 8701G/A also leads to an alanine/threonine substitution in ATPase6, the F0 subunit 6 of complex V (ATP synthase). The NADH-ubiquinone oxidoreductase contributes to the generation of the proton electrochemical gradient that is then used by the ATP synthase to drive ATP production.

Given the pivotal role of the oxidative phosphorylation (OXPHOS) cycle in metabolic processes, there are a large number of potential evolutionary explanations behind the strong correlation we observed. One hypothesis that has previously received considerable attention is a possible tradeoff between ATP synthesis and thermogenesis (Mishmar *et al*. 2003; Ruiz-Pesini *et al*. 2004). Lower oxidative phosphorylation coupling across the inner mitochondrial membrane and higher proton leakage are expected to lead to less efficient ATP synthesis but will release more heat to maintain body temperature (Brand 1990). Thus, there could be different optima in the efficiency of the OXPHOS process in different environments. This could in turn have favoured different mutations in different areas of the world (Mishmar *et al*. 2003; Ruiz-Pesini *et al*. 2004). While our results are compatible with this hypothesis, the limited functional evidence available to date does not point to higher coupling efficiency in African versus European/Arctic mitochondrial genomes (Amo & Brand 2007). However, the sample size in this experiment was small (six individuals) and the individuals were not characterized for 8701G/A and 10398G/A (Amo & Brand 2007). As 10398G is fairly common throughout Europe, it may have been present in some of the European/Arctic haplotypes analysed. Direct functional tests on 8701G/A and 10398G/A are needed before reaching any conclusion on whether the patterns we observe support the energy/heat trade-off hypothesis or whether they might have been generated by another mechanism.

As humans expanded out of Africa eventually to colonize the entire globe, they experienced new environments. For many populations, this also meant exposure to far colder climates. Smooth clines in allele frequency correlated with climatic variables have been observed for SNPs in autosomal genes involved in common metabolic disorders, such as type 2 diabetes, obesity and hypertension (Young *et al*. 2005; Hancock *et al*. 2008). These patterns have been interpreted as signatures of the stress that colder climate imposed upon human populations in the past. The parallel observation of strong correlations between two correlated mitochondrial SNPs and temperature may seem even more remarkable. Indeed, owing to the lack of recombination in mtDNA, the expected efficacy of natural selection is reduced because of potential interference between selected sites (Hill & Robertson 1966). The selective pressures imposed on mtDNA sequences may have been remarkably strong to create the correlations we observe. This suggests that the two non-synonymous polymorphisms we detected in *ND3* and *ATP6* may represent previously unrecognized important determinants of human metabolism.
